# High-Accuracy Classification of Parkinson’s Disease Using Ensemble Machine Learning and Stabilometric Biomarkers

**DOI:** 10.3390/neurolint17090133

**Published:** 2025-08-26

**Authors:** Ana Carolina Brisola Brizzi, Osmar Pinto Neto, Rodrigo Cunha de Mello Pedreiro, Lívia Helena Moreira

**Affiliations:** 1 Biomedical Engineering Postgraduate Program, Anhembi Morumbi University, São José dos Campos 12247-016, Brazil; carol.brisola.cb@gmail.com (A.C.B.B.); livia.mel@animaeducacao.com.br (L.H.M.); 2 Basic Institute of Biosciences, Taubaté University (Unitau), Taubaté 12020-040, Brazil; 3 Department of Kinesiology, California State University San Marcos (CSUSM), San Marcos, CA 92096, USA; 4 Arena235 Research Lab, São José dos Campos 12246-876, Brazil; 5 Center of Innovation Technology and Education-CITÉ, São José dos Campos 12247-016, Brazil; 6 Department of Physical Education, Estácio de Sá University, Teresópolis 25963-150, Brazil; rodrigocmp1@gmail.com

**Keywords:** Parkinson’s disease, postural sway, stabilometry, machine learning, ensemble learning, biomarkers, feature importance, center of pressure, diagnostic models, geriatric biomechanics

## Abstract

**Background**: Accurate differentiation of Parkinson’s disease (PD) from healthy aging is crucial for timely intervention and effective management. Postural sway abnormalities are prominent motor features of PD. Quantitative stabilometry and machine learning (ML) offer a promising avenue for developing objective markers to support the diagnostic process. This study aimed to develop and validate high-performance ML models to classify individuals with PD and age-matched healthy older adults (HOAs) using a comprehensive set of stabilometric parameters. **Methods**: Thirty-seven HOAs (mean age 70 ± 6.8 years) and 26 individuals with idiopathic PD (Hoehn and Yahr stages 2–3, on medication; mean age 66 years ± 2.9 years), all aged 60–80 years, participated. Stabilometric data were collected using a force platform during quiet stance under eyes-open (EO) and eyes-closed (EC) conditions, from which 34 parameters reflecting the time- and frequency-domain characteristics of center-of-pressure (COP) sway were extracted. After data preprocessing, including mean imputation for missing values and feature scaling, three ML classifiers (Random Forest, Gradient Boosting, and Support Vector Machine) were hyperparameter-tuned using GridSearchCV with three-fold cross-validation. An ensemble voting classifier (soft voting) was constructed from these tuned models. Model performance was rigorously evaluated using 15 iterations of stratified train–test splits (70% train and 30% test) and an additional bootstrap procedure of 1000 iterations to derive reliable 95% confidence intervals (CIs). **Results**: Our optimized ensemble voting classifier achieved excellent discriminative power, distinguishing PD from HOAs with a mean accuracy of 0.91 (95% CI: 0.81–1.00) and a mean Area Under the ROC Curve (AUC ROC) of 0.97 (95% CI: 0.92–1.00). Importantly, feature analysis revealed that anteroposterior sway velocity with eyes open (V-AP) and total sway path with eyes closed (TOD_EC, calculated using COP displacement vectors from its mean position) are the most robust and non-invasive biomarkers for differentiating the groups. **Conclusions**: An ensemble ML approach leveraging stabilometric features provides a highly accurate, non-invasive method to distinguish PD from healthy aging and may augment clinical assessment and monitoring.

## 1. Introduction

The advent of machine learning (ML) has significantly transformed data analysis across numerous biomedical research domains, offering powerful tools for uncovering complex patterns and building predictive models from multifaceted datasets [1]. In medicine, ML applications range from image analysis in radiology and pathology to the interpretation of genomic data, drug discovery, and the development of clinical decision support systems, ultimately aiming to enhance diagnostic accuracy, personalize treatment, and improve patient outcomes [2,3,4]. Machine learning methods are especially suitable for neurodegenerative disorders such as Parkinson’s disease, given their inherent complexity, heterogeneity, and progressive nature. ML’s capability to handle large, multidimensional datasets, recognize subtle, nonlinear patterns, and integrate multiple modalities of data (e.g., biomechanical, clinical, and imaging) offers significant potential to enhance diagnostic precision, identify early biomarkers, and support personalized therapeutic strategies [5]. This study leverages these computational advancements to address the persistent challenges associated with neurodegenerative disorders.

Parkinson’s disease (PD) is a progressive neurodegenerative disorder characterized by a combination of motor symptoms, including bradykinesia, rigidity, resting tremor, and postural instability [6,7,8]. The diagnosis of PD, particularly in its early stages, relies heavily on clinical assessment, which can be subjective and may lead to misdiagnosis, particularly when differentiating PD from other Parkinsonian syndromes or essential tremors [9,10]. Accurate and early diagnosis of PD is crucial for timely therapeutic intervention, patient management, and enrollment in clinical trials to develop neuroprotective strategies [11,12,13]. Herein, we explored the potential of machine learning (ML) in conjunction with stabilometric parameters to enhance diagnostic accuracy. Integrating machine learning with stabilometric parameters uniquely leverages the objective and sensitive quantification of subtle motor impairments, potentially allowing earlier and more precise differentiation of PD from healthy aging compared to traditional clinical assessments alone.

Quantitative assessment of motor function through biomechanical analysis offers a promising avenue for developing objective markers to support PD diagnosis and monitor disease progression. Changes in gait and balance are among PD’s earliest and most debilitating motor manifestations [14,15]. Parameters derived from postural sway, such as center-of-pressure (COP) displacement, velocity, and area, have been shown to differ between individuals with PD and healthy controls [16]. The characteristics of these measures, including stride length, gait speed, cadence, and variability, are often altered in patients with early PD [17]. These objective and quantifiable measures may provide sensitive indicators of underlying motor dysfunction that are not always apparent in standard clinical examinations.

Machine learning techniques have emerged as powerful tools for analyzing complex and high-dimensional datasets, such as those derived from biomechanical assessments. By learning patterns from feature sets, ML algorithms can build classification models that can distinguish between different groups with high accuracy [18]. ML has been increasingly applied in the context of PD to differentiate patients with PD from healthy controls using various data modalities, including gait [14,19,20], postural sway [1], speech [9,21,22], and handwriting [23]. These approaches can enhance diagnostic accuracy, provide objective support for clinical decisions, and identify subtle motor signatures that may precede the onset of clinical symptoms.

A growing body of literature has explored the use of ML with stabilometric data to assess PD. For instance, studies have employed Support Vector Machines (SVMs), Random Forests (RFs), and Neural Networks, among other algorithms, to classify patients with PD from healthy controls [24,25]. Standard input features include time-domain parameters (e.g., sway area, velocity, RMS of COP displacement) and frequency-domain parameters (e.g., power spectral density at different frequencies derived from COP trajectories [26,27,28]. While many studies report promising classification accuracies, performance can vary based on dataset size, patient characteristics (e.g., disease stage, medication status), specific stabilometric parameters used, and the rigor of ML validation. Furthermore, direct comparisons between studies are often hindered by methodological differences, and there remains a need for models validated with robust techniques, such as bootstrap procedures, to ensure generalizability and provide confidence intervals for the performance metrics. Some studies may also focus on limited feature sets or may not fully explore the potential of ensemble classifiers, which can often improve stability and accuracy over individual model processes [2,3,9,22,26,29]. Our study addresses these limitations by utilizing a comprehensive and rigorously validated stabilometric feature set combined with a robust ensemble machine learning model validated through repeated bootstrapping. This approach ensures both methodological rigor and generalizability of the findings, providing a more reliable classification framework than previously reported studies.

The primary aim of this study was to develop and evaluate machine learning models capable of accurately differentiating individuals with Parkinson’s disease (PD) from age-matched Healthy Older Adults (HOAs) based on a comprehensive set of biomechanical parameters derived from quiet stance postural sway analysis. We hypothesized that a data-driven approach leveraging tuned ensemble classifiers could achieve high diagnostic accuracy and provide insights into the most discriminative biomechanical features. Such a tool could be an objective aid in the clinical assessment of individuals suspected of having PD. We specifically employed tuned ensemble classifiers because they combine diverse algorithmic strengths, mitigate individual model biases, and enhance generalization. Such ensemble approaches not only optimize prediction accuracy but also offer greater interpretability of feature relevance by identifying robust stabilometric parameters that are consistently associated with PD. Beyond providing an objective diagnostic aid, these insights have meaningful clinical implications, potentially informing therapeutic strategies and early interventions by identifying specific impairments in postural control. The novelty of this study lies in the integration of a broad, physiologically informed stabilometric feature set with rigorous ensemble machine learning and extensive validation. This approach enables objective, quantitative differentiation of PD from healthy aging and highlights the most clinically relevant sway parameters using both statistical and machine learning criteria.

## 2. Materials and Methods

### 2.1. Study Design, Setting, and Population

This study was approved by the local Research Ethics Committee of Anhembi Morumbi University, São Paulo, Brazil (CAAE: 24205419.8.0000.5492 approval on 6 March 2020, and CAAE: 08957419.0.000.5492 approval on 24 March 2019). All participants provided written informed consent before their inclusion in the study. Individuals aged 60–80 years were recruited for this study and divided into two groups: the HOA Group (Healthy Older Adults): 37 healthy older adults (30 women and 7 men); and the PD Group (PD): 26 older adults (10 women, 16 men) diagnosed with idiopathic Parkinson’s disease.

The inclusion criteria for both groups were the absence of lesions and/or ulcerations in the lower limbs, non-regular use of alcoholic beverages, and non-smoking status. Subjects with any functional impairment unrelated to PD (for the PD group) that could interfere with the assessments were excluded from the study. Individuals with significant visual impairment or a diagnosis of vestibular disease were excluded from the study following health screening. Participants who used corrective lenses wore them throughout the evaluation. The exclusion criteria included significant uncorrected visual impairment, vestibular disorders (including benign paroxysmal positional vertigo, or BPPV), recent falls, primary orthopedic conditions affecting the lower limbs, and the use of assistive devices. All current medications were reviewed for adverse effects related to the balance. The predominance of women in the sample reflects the demographic profile of the participants at the senior center. All participants were regularly monitored for their health conditions, and no cases of gynecological disorders affecting balance were identified.

Individuals with PD met the UK Parkinson’s Disease Society Brain Bank criteria for the diagnosis of idiopathic PD [30]. Participants with PD were included if they were classified as Hoehn and Yahr stages 2 or 3 [31], consistent with established research practice, and did not have other associated neurological or orthopedic diseases that could significantly affect mobility (Table 1). Subgroup analysis by stage was not performed because of the limited sample size. At the time of recruitment, all patients with PD were undergoing treatment with levodopa and/or with dopamine agonists.

All individuals with PD were assessed in the ‘on’ phase of their usual levodopa regimen, typically 1–2 h after the morning dose, to reduce confounding from severe motor fluctuations. Notably, previous studies have indicated that levodopa has a limited influence on postural stability in patients with PD [32,33]. Chronic conditions, such as diabetes and controlled cardiac disease, were present in some healthy older adults. All patients were under regular medical supervision and clinically stable, with no acute symptoms at the time of testing.

### 2.2. Clinical and Anthropometric Assessments

Before the biomechanical testing, all participants underwent an initial evaluation that included the collection of anthropometric data (weight [kg], height [m], and calculation of body mass index [BMI] [kg/m^2^]) and a detailed health history (including pre-existing diseases and medications in use) (Table 1). All participants confirmed that they had eaten before the assessments. Blood pressure was measured with the participants seated and at rest, both before and after the assessment protocol, using standard clinical procedures.

All participants with PD were assessed using the Movement Disorder Society-Unified Parkinson’s Disease Rating Scale (MDS-UPDRS) Part III (motor examination) [34], which included specific queries regarding the presence and impact of tremors, dyskinesias, and dystonias. Individuals whose involuntary movements might have confounded the assessment of postural sway were excluded.

Although a formal cognitive assessment was not performed, participants underwent informal cognitive screening during the informed consent and instructional procedures. Individuals who demonstrated difficulty in comprehending the task instructions or consenting procedures were excluded to ensure the reliability of the stabilometric data collection. Furthermore, the participants’ functional capacity and self-care were evaluated using the Barthel Index. This index assesses an individual’s ability to perform activities of daily living, functional mobility, and gait, and provides a quantitative measure of functional independence [35,36]. The Barthel Index was selected for its extensive clinical validation, relevance in assessing functional autonomy, and established adaptation to the Brazilian Portuguese language. Although more recent ADL instruments exist, the Barthel Index remains the recommended choice by expert panels for neurological rehabilitation [37]. All participants in the HOA group scored 100 on the Barthel Index.

### 2.3. Stabilometric Assessment (Equipment)

To accurately capture postural sway abnormalities characteristic of PD, we employed a detailed stabilometric data collection protocol. Stabilometric assessment was performed using an S-PLATE force platform (Medicapteurs, Balma, France) that was 610 mm wide and 580 mm deep, with an active area of 400 × 400 mm. The platform contained 1600 resistive sensors, each measuring 0.64 cm^2^, allowing for a detailed analysis of the center-of-pressure (COP) oscillation. The COP data acquisition frequency (Fs) was 10 Hz.

### 2.4. Stabilometric Data Collection

For stabilometric data collection, participants were instructed to remain barefoot in an orthostatic position on the force platform, with their feet comfortably shoulder-width apart within the sensitive area of the platform. The upper limbs were kept alongside the body, and the participants were instructed to maintain a fixed gaze on a red circular target positioned 1 m away from eye level. Data collection was performed under two conditions: Eyes Open (EO), where participants kept their eyes open and maintained a fixed gaze on the target, and Eyes Closed (EC), where participants closed their eyes while performing the task. Each condition lasted 15 s, with no rest period between conditions, and only one trial was recorded for each condition.

### 2.5. Stabilometric Variable Extraction

Raw COP data (anteroposterior [AP] and mediolateral [ML] coordinates over time) were processed using custom routines in MATLAB (R2022a; The MathWorks Inc., Natick, MA, USA). The raw AP and ML COP signals were filtered using a 4th-order Butterworth bandpass filter with cut-off frequencies of 0.005–4.9 Hz, and linear trends were removed from the filtered signals.

From the processed and detrended center-of-pressure (COP) data, we calculated 17 stabilometric variables (11 time-domain variables; 6 frequency-domain variables) for each condition, eyes open and eyes closed, resulting in a total of 34 features for use in the machine learning analysis. These parameters were selected based on their theoretical and empirical relevance, allowing us to capture visual feedback-related adaptations in postural sway [27,38].

The variables included the following:

Time-domain variables (11):

- Total oscillation displacement (TOD) (mm): calculated as the sum of the magnitudes of the COP displacement vectors from the mean COP position at each sampled time point).
- Standard Deviation of COP displacement in AP and ML directions (Std-AP, Std-ML, mm).
- Root Mean Square of COP displacement in the AP and ML directions (RMS-AP, RMS-ML, mm).
- Displacement Amplitude (Peak-to-peak) of COP in AP and ML directions (DA-AP, DA-ML, mm).
- The mean Velocity of COP in AP and ML directions (V-AP, V-ML, mm/s): calculated as the sum of absolute differences between successive COP points in each direction, multiplied by the sampling frequency and divided by the number of displacement intervals.
- Total Mean Velocity of COP (V-T, mm/s): calculated as the sum of the magnitudes of 2D displacement vectors between successive COP points, multiplied by the sampling frequency, and divided by the number of displacement intervals.
- Area of the 95% confidence ellipse (area, mm^2^): calculated from the covariance matrix of the AP and ML COP displacement.

Frequency-domain variables were derived from the fast Fourier transform (FFT). The power spectral density was calculated for both AP and ML COP signals. The power (mm^2^/Hz) was then summed within the following frequency bands: Low Frequency (LF): 0.01–0.1 Hz (LF-AP, LF-ML); Medium Frequency (MF): >0.1–0.5 Hz (MF-AP, MF-ML); and High Frequency (HF): >0.5–1.0 Hz (HF-AP, HF-ML).

The complete list of 34 features included: TOD, Std-AP, Std-ML, RMS-AP, RMS-ML, DA-AP, DA-ML, V-AP, V-ML, Area, V-T, LF-AP, MF-AP, HF-AP, LF-ML, MF-ML, HF-ML, and their corresponding counterparts for the Eyes Closed (EC) condition (e.g., TOD_EC, Std-AP_EC, etc.).

### 2.6. Machine Learning Data Analysis

The primary objective of the machine learning analysis was to develop and validate classification models using the extracted stabilometric variables to distinguish between individuals in the HOA and PD groups.

Data Preprocessing for Machine Learning: The 34 stabilometric variables were compiled into a dataset. Before model training, all features were standardized (scaled to have a zero mean and unit standard deviation). This scaling was applied within each cross-validation fold during hyperparameter tuning and within each training set of the bootstrap iterations to prevent data leakage from the test set.

Data Splitting and Model Validation Strategy: For initial hyperparameter tuning, the dataset (HOA and PD samples) was divided into a main training set (70%) and a main test set (30%) using stratified splitting to maintain the proportion of HOAs and PD individuals in both sets. Hyperparameter optimization was performed on the main training set using cross-validation. The final reported model performance was derived using 15 iterations of stratified train–test splits (70% train and 30% test) and an additional bootstrap procedure of 1000 iterations to derive reliable 95% confidence intervals.

### 2.7. Hyperparameter Tuning

Three classification algorithms—Random Forest (RF), Gradient Boosting (GB), and Support Vector Machine (SVM)—were selected for evaluation owing to their proven efficacy in biomedical classification tasks and their different approaches to learning decision boundaries. RF and GB are ensemble methods known for their robustness and ability to handle high-dimensional data [39,40]. SVMs are powerful for finding optimal separating hyperplanes, especially in complex, nonlinear feature spaces when appropriate kernels are used [41]. For each algorithm, GridSearchCV with 3-fold cross-validation was performed on the main training set (70% of the HOA and PD data) to identify the optimal combination of hyperparameters. Grid search was selected for its transparency, reproducibility, and proven effectiveness in optimizing models with limited hyperparameter space. Although alternative approaches, such as population-based optimization algorithms, may offer advantages in more complex settings, the grid search was computationally efficient. It yielded stable results for our dataset and model set. Future studies in larger or more complex settings could benefit from benchmarking these methods. The metric used for optimization in GridSearchCV was the Area Under the ROC Curve (AUC ROC) for binary classification, as it provides a comprehensive measure of a classifier’s performance across all classification thresholds. SMOTE was included as a step within the pipeline for GridSearchCV to address class imbalance during the training of each fold.

The parameter grids explored for each model were as follows: for Random Forest, the tuned parameters included n_estimators (100, 150), max_depth (None, 20, 30), min_samples_split (2, 5, 10), and min_samples_leaf (1, 2, 4); For Gradient Boosting, parameter tuning included n_estimators (100, 150), learning_rate (0.01, 0.1, 0.15), max_depth (4, 6, 8), subsamples (0.7, 0.9), min_samples_split (2, 6), and min_samples_leaf (1, 3); and for the SVM, the tuned parameters included C (1, 10, 50), kernel (rbf, linear, poly), gamma (scale, auto, 0.1), and degree (2, 3) for the polynomial kernel. Using the available computational resources, the tuning process durations were approximately 35.8 s for Random Forest, 47.5 s for Gradient Boosting, and 5.5 s for SVM.

### 2.8. Class Imbalance Handling

The dataset showed a moderate class imbalance (HOA *n* = 37, PD *n* = 26; ratio ≈ 1.42:1). To assess any impact of imbalance on model behavior, we ran the complete pipeline with and without Synthetic Minority Over-sampling Technique (SMOTE). When used, SMOTE was applied only to the training partition inside each cross-validation fold during hyperparameter tuning and inside each bootstrap split at evaluation; test sets were never augmented. This design prevents leakage while allowing a direct, paired comparison of performance with vs. without oversampling.

### 2.9. Ensemble Model Construction

A VotingClassifier ensemble model was constructed by combining three individual classifiers (RF, GB, and SVM) using their respective optimized hyperparameters, identified through GridSearchCV. Soft voting, which averages the predicted probabilities from each base model, was employed to make the final classification decision.

Bootstrap Validation and Performance Metrics: To rigorously assess the robustness and stability of the optimized models (RF, GB, SVM, and Ensemble), we first conducted 15 iterations of stratified train–test splits (70% train and 30% test). For each split, the entire preprocessing pipeline, including scaling and feature transformations, was independently applied. Performance metrics were calculated for each test set, after which an additional bootstrap procedure of 1000 iterations was employed on these results to derive reliable 95% confidence intervals for accuracy, F1-score, AUC ROC, sensitivity, and specificity. Fifteen iterations were selected as a pragmatic balance between achieving a robust model evaluation and maintaining computational feasibility. Preliminary analyses demonstrated that this number provided stable estimates of the model performance with minimal variability between iterations.

To formally compare pipelines with and without SMOTE under identical resampling, we evaluated both approaches across the same 15 stratified 70/30 train–test splits (paired by random seed). For each split and model (RF, GB, SVM), we computed AUC, accuracy, F1 (positive/negative class), sensitivity, and specificity, and applied two-sided paired Wilcoxon signed-rank tests with Benjamini–Hochberg false discovery rate correction across metrics and models. We also assessed the concordance of feature importance ranks between pipelines for RF and GB using Spearman correlation. In rare cases where all paired differences were zero, the Wilcoxon statistic is undefined; these entries were treated as NA and do not affect the conclusions.

### 2.10. Feature Importance Analysis

Given the number of stabilometric features (*n* = 34) relative to the moderate sample size, explicit feature selection procedures (e.g., filter or wrapper methods) were considered during preliminary analyses. However, owing to the inherent feature-selection capabilities of Random Forest and Gradient Boosting models, both of which automatically prioritize features by importance, we opted to rely on their embedded feature-selection mechanisms, thus preserving model interpretability and methodological simplicity. For the tree-based models (Random Forest and Gradient Boosting), feature importance (derived from the mean decrease in impurity or similar measures) was extracted from models trained on the training data from the first data split. This analysis aimed to identify the stabilometric variables that contributed most significantly to the discrimination between the HOA and PD groups.

**Figure 1:**
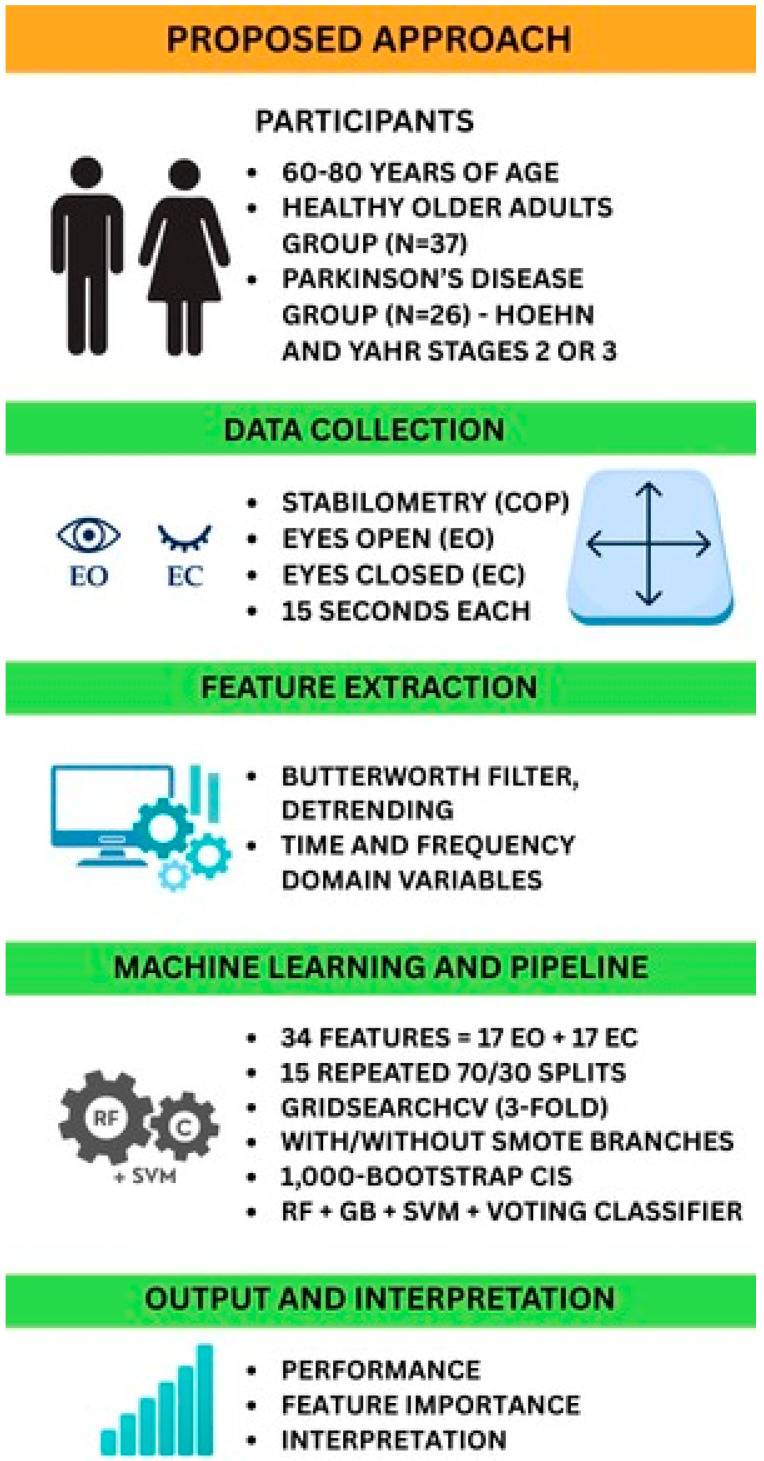
Workflow of the proposed approach. Abbreviations: COP, center of pressure; EO, eyes open; EC, eyes closed; SMOTE, Synthetic Minority Over-sampling Technique; CIs, confidence intervals; RF, random forest; GB, gradient boosting; SVM, support vector machine.

Figure 1 provides a schematic of the end-to-end analytical pipeline, from stabilometric data acquisition through final model interpretation. This diagram illustrates each processing stage: data acquisition, preprocessing, feature extraction, statistical analysis, model training and validation, and output interpretation, enabling readers to visualize and reproduce our methodology.

### 2.11. Statistical Analysis of Group Characteristics and Stabilometric Parameters

Descriptive statistics for participant characteristics (age, sex, BMI, Barthel Index scores, and MDS-UPDRS Part III scores) were also calculated for the PD group. Continuous variables are presented as mean ± standard deviation (SD) or median (with Interquartile Range [IQR]) based on their distribution, as assessed by the Shapiro–Wilk test (Table S2). Categorical variables are presented as counts and percentages. Differences in baseline characteristics between the HOA and PD groups were assessed using independent sample t-tests or Mann–Whitney U tests for continuous variables and chi-squared tests or Fisher’s exact test for categorical variables.

Univariate statistical tests were conducted for 34 stabilometric parameters to compare the HOA and PD groups. The normality of each parameter within each group was assessed using the Shapiro–Wilk test (Supplementary Table S2), and the homogeneity of variances was evaluated using Levene’s test. Based on these assessments, the Mann–Whitney U test was used for group comparisons. To account for multiple comparisons across the 34 parameters, *p*-values were adjusted using the Bonferroni correction and False Discovery Rate (FDR) control via the Benjamini–Hochberg procedure. Differences were considered statistically significant at an adjusted *p*-value of less than 0.05. The median [IQR] values for each parameter are reported for both groups.

Owing to the number of dependent variables (34 parameters) relative to the sample size in the PD group (*n* = 26), Multivariate Analysis of Variance (MANOVA) was deemed inappropriate and therefore not performed. The primary assessment of the model’s classification performance relied on metrics derived from the bootstrap validation procedure.

## 3. Results

### 3.1. Participant Characteristics

The analysis included 37 healthy older adults (HOAs) and 26 individuals with Parkinson’s disease (PD). Table 1 summarizes the participants’ demographic and clinical characteristics.

### 3.2. Statistical Analysis of Stabilometric Parameters

Univariate statistical analysis was performed on 34 stabilometric parameters to compare the HOA and PD groups. After applying the Benjamini–Hochberg False Discovery Rate (FDR) correction for multiple comparisons, 11 parameters showed statistically significant differences (FDR *p* < 0.05) between the groups. The Bonferroni correction, a more conservative approach, identified two of these parameters as significant (Bonferroni *p* < 0.05). Descriptive statistics (median [IQR]) for all parameters are presented in Table 2, and the results of the univariate tests are presented in Supplementary Table S1. Both False Discovery Rate (FDR) and Bonferroni corrections were applied to address multiple comparisons, each offering distinct trade-offs. While the FDR correction controls the expected proportion of false positives and preserves higher sensitivity, the Bonferroni approach provides a more conservative method by strictly controlling the family-wise error rate, thereby enhancing specificity at the cost of sensitivity. Reporting both allows for a comprehensive understanding of significant differences, striking a balance between statistical rigor and practical interpretability.

**Figure 2:**
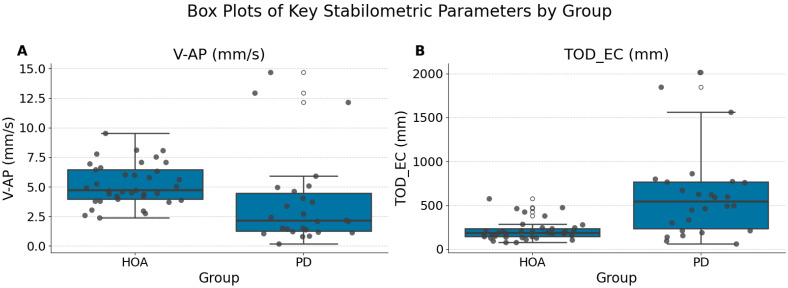
Box plots of key significantly different stabilometric parameters between the HOA and PD groups. (**A**) Anteroposterior sway velocity with eyes open (V-AP (mm/s) (EO)). (**B**) Total Oscillation Displacement with eyes closed (TOD_EC (mm) (EC)). Boxes represent the interquartile range (IQR), the horizontal line within the box indicates the median, and whiskers extend 1.5 times the IQR. The individual data points are overlaid. Significant differences (Bonferroni, *p* = 0.0078 for V-AP and *p* = 0.0011 for TOD_EC) were observed for both parameters.

The parameters that remained significant after Bonferroni correction were anteroposterior sway velocity with eyes open (V-AP (mm/s) (EO)) (PD Median 2.14 [IQR 3.24] vs. HOA Median 4.73 [IQR 2.48]; Bonferroni *p* = 0.0078) and total sway path during eyes-closed conditions (TOD_EC (mm) (EC)) (PD Median 542.74 [IQR 531.14] vs. HOA Median 186.89 [IQR 90.65]; Bonferroni *p* = 0.0011). Individuals with PD exhibited significantly lower median V-AP (mm/s) (EO) and significantly greater median TOD_EC (mm) (EC) than HOA participants (Figure 2). Specifically, individuals with Parkinson’s disease exhibited significantly lower anteroposterior (AP) sway velocity under eyes-open conditions, possibly reflecting increased postural stiffness or bradykinetic adjustments typical of motor impairments associated with PD. Conversely, the significantly greater total sway path observed under eyes-closed conditions indicates impaired sensory integration and heightened reliance on visual input for postural stability in patients with PD, highlighting compromised proprioceptive or vestibular processing.

The additional nine parameters found to be significantly different after FDR correction included Std-AP (mm) (EO), RMS-AP (mm) (EO), DA-AP (mm) (EO), V-T (mm/s) (EO), LF-AP (mm^2^/Hz) (EO), MF-AP (mm^2^/Hz) (EO), Std-AP_EC (mm) (EC), RMS-AP_EC (mm) (EC), and DA-AP_EC (mm) (EC) (all FDR *p* < 0.05). These differences predominantly highlighted alterations in anteroposterior sway control and overall sway magnitude in the eyes-closed condition in the PD group compared with the HOA group. For instance, participants with PD exhibited lower median values for most eyes-open AP sway magnitude and velocity parameters but higher median values for eyes-closed AP sway magnitude parameters and TOD_EC (mm). Clinically, these findings indicate that patients with PD show reduced magnitude and variability of anteroposterior sway in the eyes-open condition, reflecting increased rigidity and reduced adaptability in the quiet stance. Under eyes-closed conditions, elevated values for sway amplitude and variability suggest impaired use of non-visual sensory inputs. Collectively, these metrics reinforce the notion that PD involves both hypo-kinetic and sensory-dependent postural control strategies, further distinguishing patients with PD from healthy older adults.

### 3.3. Performance of Machine Learning Models

Given the modest imbalance (ratio ≈ 1.42:1), we re-ran the complete analysis with and without SMOTE. Across the same 15 paired stratified splits, SMOTE and non-SMOTE pipelines performed equivalently for all models. Paired Wilcoxon signed-rank tests found no significant differences in AUC, accuracy, F1, sensitivity, or specificity after FDR correction (all q ≥ 0.22; Supplementary Table S3). Importantly, feature-importance rankings were highly concordant between pipelines (Random Forest: Spearman ρ = 0.80, *p* < 0.0001; Gradient Boosting: ρ = 0.77, *p* < 0.0001), and the top contributors remained the same. Given this parity, we present non-SMOTE metrics in the main text and include SMOTE data in the Supplementary Materials (Table S4).

The best hyperparameters obtained for each model were as follows: RF: n_estimators = 100, max_depth = None, min_samples_split = 2, min_samples_leaf = 2, GB: n_estimators = 150, learning_rate = 0.01, max_depth = 4, min_samples_split = 6, min_samples_leaf = 1, subsample = 0.7, SVM: kernel = ‘rbf’, C = 1, degree = 2, gamma = 0.1. Following hyperparameter optimization, four machine learning models—RF, GB, SVM, and Ensemble Voting Classifier—were evaluated for their ability to differentiate between HOAs and individuals with PD. The performance metrics derived from the 15 iterations of bootstrap validation are summarized in Table 3.

The ensemble voting model demonstrated the best overall performance. It achieved a mean accuracy of 0.9053 (95% CI: 0.8079–1.0000) and a mean Area Under the ROC Curve (AUC ROC) of 0.9727 (95% CI: 0.9244–1.0000). For the identification of PD (considered the positive class), the ensemble yielded a mean sensitivity (recall) of 0.8833 (95% CI: 0.7500–1.0000) and a mean specificity (recall for HOAs) of 0.9212 (95% CI: 0.8182–1.0000). The mean F1-score for PD was 0.8867 (95% CI: 0.7675–1.0000), and for HOAs, it was 0.9181 (95% CI: 0.8318–1.0000).

The individually tuned models also exhibited strong performances. The Tuned Gradient Boosting model achieved a mean accuracy of 0.9018 (95% CI: 0.8421–1.0000) and a mean AUC-ROC of 0.9652 (95% CI: 0.9205–1.0000). The Tuned SVM yielded a mean accuracy of 0.8772 (95% CI: 0.7895–0.9474) and a mean AUC ROC of 0.9591 (95% CI: 0.9017–1.000). The Tuned Random Forest model resulted in a mean accuracy of 0.8912 (95% CI: 0.8079–0.9474) and a mean AUC ROC of 0.9568 (95% CI: 0.8648–1.0000).

**Figure 3:**
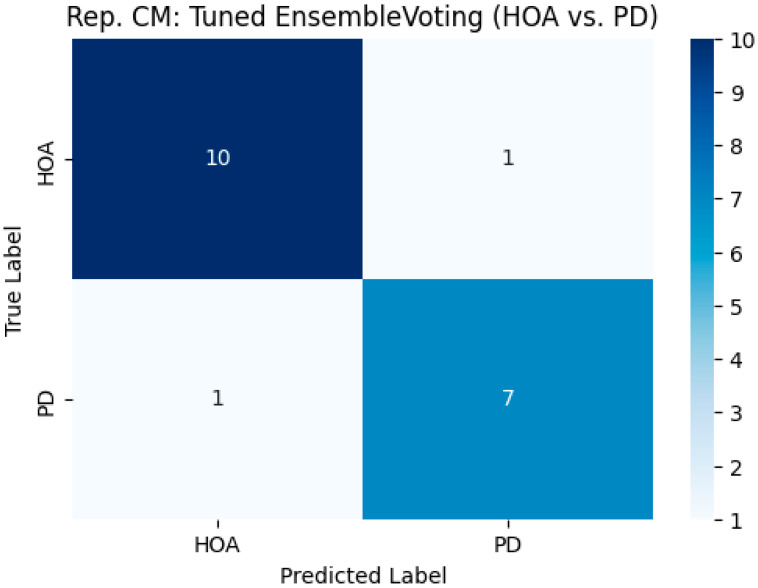
Representative confusion matrix for the Tuned Ensemble Voting model, classifying healthy older adults (HOAs) and Parkinson’s disease (PD) patients from the first bootstrap run.

The classification performance of the tuned ensemble voting model, as observed in a representative (first split) run, is presented in Table 4, along with the confusion matrix shown in Figure 3. In a representative run, the ensemble model achieved an accuracy of 0.8947. The precision of the PD class was 0.8750, the recall (sensitivity) was 0.8750, and the F1-score was 0.8750. The HOA class achieved a precision of 0.9091, a recall (specificity for PD) of 0.9091, and an F1-score of 0.9091. The confusion matrix (Figure 3) for this run indicated that out of the 19 test samples, one HOA individual was misclassified as having PD, and one individual with PD was misclassified as a HOA. The representative runs for the tuned RF and SVM models yielded confusion matrices and performance metrics identical to those of the ensemble in this specific first bootstrap iteration. In contrast, the tuned GB model misclassified two HOA individuals as individuals with PD and one PD individual as a HOA in its representative run (accuracy: 0.8421).

### 3.4. Feature Importances

To improve the interpretability of our tree-based models, we examined the relative feature importances from representative runs of the tuned Random Forest and Gradient Boosting classifiers. Importantly, no feature selection was performed before model training; all 34 stabilometric parameters (17 under eyes-open [EO] and 17 under eyes-closed [EC] conditions) were included in each model.

**Figure 4:**
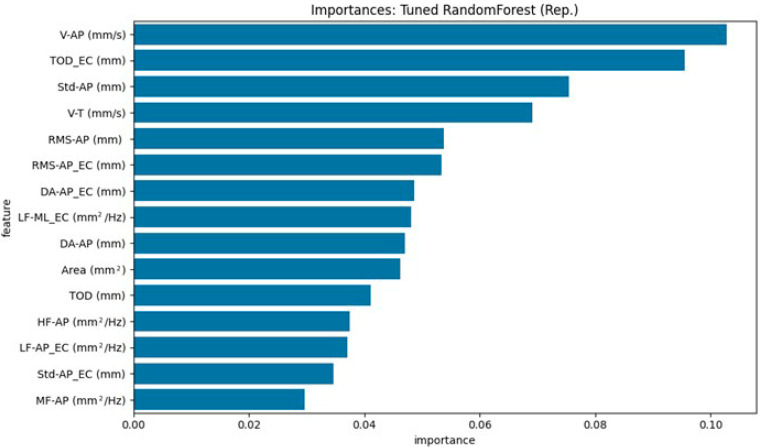
Top 15 stabilometric features identified by the Tuned Random Forest model (representative run) for distinguishing HOAs from PD.

**Figure 5:**
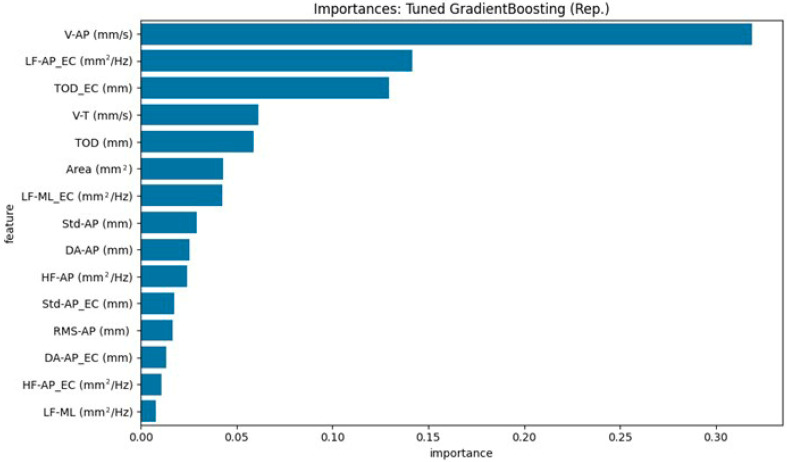
Top 15 stabilometric features identified by the Tuned Gradient Boosting model (representative run) for distinguishing HOAs from PD.

Figure 4 and Figure 5 illustrate the top 15 ranked features, based on each model’s internal importance metric. For ease of interpretation, we summarize the top five contributing features in the text below. These rankings vary between models due to differences in how feature importance is calculated and how each model leverages the available information.

For the Tuned Random Forest (Figure 4), the top three contributing features were: V-AP (mm/s) [EO], TOD_EC (mm) [EC], and Std-AP (mm) [EO].

For the Tuned Gradient Boosting model (Figure 5), the top three most important features were: V-AP (mm/s) [EO], LF-AP_EC (mm^2^/Hz) [EC], TOD_EC (mm) [EC]. 

Both models consistently highlighted sway velocity and the overall magnitude of oscillation, particularly in the anteroposterior direction, as relevant features for distinguishing between PD and HOA participants. These findings are valuable for understanding the physiological underpinnings of the classification and support the relevance of these parameters in assessing postural control.

## 4. Discussion

This study aimed to develop and validate machine learning models that accurately distinguish individuals with Parkinson’s disease (PD) from age-matched Healthy Older Adults (HOAs) using a comprehensive set of stabilometric parameters. These findings demonstrate that this differentiation is feasible and highly efficacious. Our tuned ensemble voting classifier, which combines the strengths of Random Forest, Gradient Boosting, and Support Vector Machine models, emerged as the top-performing approach. An important strength of our approach is model interpretability: the use of tree-based models such as Random Forest and Gradient Boosting allowed us to extract and visualize feature importance, clearly identifying the stabilometric parameters most relevant for distinguishing Parkinson’s disease from healthy controls. This transparency not only supports the robustness of our findings but also facilitates clinical translation by highlighting the physiologically meaningful markers. Through robust bootstrap validation, the ensemble model achieved a mean accuracy of approximately 0.9053 (95% CI: 0.8421–1.0000) and an outstanding mean Area Under the ROC Curve (AUC ROC) of 0.9735 (95% CI: 0.9318–1.0000). Notably, it demonstrated a mean sensitivity of approximately 91.7% for identifying PD and a mean specificity of approximately 89.7% (i.e., correctly classifying HOAs). The individually tuned models, particularly Gradient Boosting and SVM, also exhibited excellent discriminative capabilities, with mean AUC-ROC scores exceeding 0.95. Feature importance analysis consistently highlighted parameters related to anteroposterior sway velocity and overall sway magnitude, particularly under eyes-closed conditions, as key differentiators between the two groups.

The high classification performance achieved in this study, particularly by the ensemble model, aligns favorably with, and in some aspects surpasses, the findings of previous studies employing machine learning for PD diagnosis based on motor assessments. For instance, studies utilizing gait parameters and various classifiers have reported an accuracy in the 80–95% range for differentiating PD from healthy controls [14,19]. Similarly, analyses of postural sway data have yielded comparable discrimination, with AUC values often exceeding 0.85 [28,38]. The mean AUC-ROC of 0.97 achieved by our ensemble model suggests a powerful discriminative ability of the selected stabilometric features when processed through an optimized machine learning pipeline. This level of performance highlights the potential of instrumented postural assessment, coupled with advanced analytics, to provide objective markers that could significantly aid in the clinical differentiation of PD from healthy aging individuals. The consistent identification of anteroposterior sway characteristics and eyes-closed sway magnitude as important features also resonates with the established literature on postural deficits in PD [15,16]. Our superior classification performance reflects several key aspects of our proposed methodology. First, the ensemble approach, which integrates Random Forest, Gradient Boosting, and Support Vector Machine models, capitalizes on the complementary algorithmic strengths of these models and reduces model-specific bias.

Furthermore, support vector machines, random forests, and gradient boosting were selected because of their established effectiveness in biomedical classification, suitability for moderate-sized datasets, and capacity for transparent feature importance analysis. These models represent a balance between predictive power and interpretability, which is essential for clinical translation. Second, our feature set was deliberately broad and informed by prior research, capturing both time- and frequency-domain stabilometric parameters that are sensitive to postural control deficits in patients with Parkinson’s disease. Finally, the use of repeated stratified data splits and extensive bootstrap resampling for confidence interval estimation ensured robust and generalizable results, thereby minimizing the risk of overfitting. For example, our identification of V-AP (anteroposterior sway velocity, mm/s) and TOD_EC (total sway path during eyes-closed condition, mm) as critical features is consistent with established postural phenotypes in PD, and the rigorous validation framework underpins the reliability of our findings.

Beyond classification performance, univariate statistical analysis revealed significant differences in stabilometric parameters between the PD and HOA groups, even after correction for multiple comparisons. The reduction in anteroposterior sway velocity (V-AP, mm/s) under eyes-open conditions in the PD group likely reflects the underlying pathophysiological mechanisms, such as increased axial rigidity and bradykinesia, which are hallmarks of Parkinsonian motor impairment. Based on the median values, individuals with PD exhibited significantly lower anteroposterior sway velocity (V-AP (mm/s) (EO): PD Median 2.14 [IQR 3.24] vs. HOA Median 4.73 [IQR 2.48]) and significantly greater total sway path during eyes-closed conditions (TOD_EC (mm): PD Median 542.74 [IQR 531.14] vs. HOA Median 186.89 [IQR 90.65]) than HOA participants. These observations are consistent with those reported in the literature [42, 43]. While some studies have reported increased sway velocities in PD, others, particularly those focusing on specific aspects or subgroups, have found reduced velocity in certain contexts, potentially reflecting bradykinesia or a more “rigid” postural strategy [16,44]. Patients with PD may consciously or unconsciously adopt a more ‘stiff’ or conservative strategy, even when visual feedback is available, to prevent instability or falls. This hypokinetic postural adjustment may be further exacerbated by subtle episodes of freezing or impaired anticipatory postural adjustments, ultimately resulting in reduced sway velocity during quiet standing. These findings emphasize that diminished postural dynamism, even under seemingly optimal conditions, can serve as an early and objective marker of PD-related balance dysfunction. The finding of an increased total sway path with eyes closed (TOD_EC (mm)) strongly aligns with reports of increased postural instability and greater reliance on visual feedback in PD [45,46]; patients with PD often exhibit larger sway when visual cues are removed. While direct comparison of absolute median values across studies can be challenging due to variations in equipment, recording protocols, and patient characteristics (e.g., disease duration, medication status), the pattern of increased overall sway under eyes-closed conditions in our PD cohort mirrors the established findings.

Feature importance analysis from our machine learning models, particularly the Random Forest model, further reinforced the relevance of these statistically significant parameters. Notably, V-AP (mm/s) (Eyes Open) and TOD_EC (mm) (Eyes Closed) were identified as the most important features for distinguishing PD from HOAs. This convergence between statistical significance in univariate tests and high importance in a multivariate classification model strengthens the argument that these specific aspects of postural control are robustly altered in patients with PD. The lower anteroposterior sway velocity (V-AP (EO)) in the PD group observed in our study might reflect aspects of Parkinsonian motor control, such as increased stiffness or a more cautious postural strategy during a quiet stance with the eyes open. This finding contrasts with some reports of generally higher sway velocities in PD, suggesting that the specific context (eyes open, anteroposterior direction) and characteristics of the PD cohort (e.g., Hoehn and Yahr stage and medication status) may influence this parameter. The increased total sway path with eyes closed (TOD_EC (EC)) underscores the greater dependence on visual cues in PD and the compromised ability to effectively utilize proprioceptive and vestibular information for postural stabilization when vision is occluded [47]. Identifying these specific parameters using our models not only supports their pathophysiological relevance but also highlights their potential utility as components for an objective, quantitative assessment of PD.

The convergence between univariate statistical significance and the high feature importance rankings assigned by our machine learning models provides particularly robust evidence for the clinical relevance of parameters such as V-AP (mm/s) and TOD_EC (mm). When independently significant features are consistently prioritized by multivariate models, it suggests that these parameters are both statistically reliable and central to accurate discrimination in real-world, multidimensional data, bolstering confidence in their diagnostic value.

These stabilometric features are considered ‘objective’ because they are derived from direct biomechanical measurements obtained using force platforms, minimizing subjectivity or examiner bias. Their ‘quantitative’ nature comes from precise, continuous measurement (in mm or mm/s) of motor output, enabling reproducible assessments and facilitating standardized monitoring of disease progression or intervention response in clinical settings.

### 4.1. Clinical Implications

The ensemble ML model developed in this study provides clinicians with a robust, non-invasive diagnostic tool capable of objectively differentiating patients with PD from healthy aging individuals. The highlighted parameters, anteroposterior sway velocity (EO) and total sway path (EC), can be readily integrated into routine clinical assessments, offering the potential for early identification and management of PD, thus facilitating timely therapeutic intervention. In addition to supporting diagnostic differentiation, quantitative stabilometric assessment, particularly when paired with machine learning, holds promise for the longitudinal monitoring of treatment effectiveness or disease progression. By enabling the detection of subtle changes in postural control, this approach could potentially identify early signs of deterioration or therapeutic response even before overt clinical symptoms emerge, supporting more proactive and personalized management strategies.

### 4.2. Limitations and Future Directions

While the modest sample size reflects a practical limitation of single-center clinical research, we mitigated the risks of overfitting and instability through repeated stratified splitting and bootstrap resampling for all metrics. We recognize that future studies in larger, independent cohorts are essential for further validation and broader generalizability.

Future research should explore the utility of this ML-driven stabilometric analysis across different stages of PD severity and various medication states (“on” vs. “off” phases). Longitudinal studies investigating the ability of this model to track disease progression and therapeutic responses will further extend its clinical utility.

An important translational goal is the development of user-friendly clinical software tools that seamlessly integrate stabilometric analysis and machine learning-based decision support, thereby facilitating widespread adoption in routine neurological practice.

## 5. Conclusions

This study successfully demonstrated that a machine learning approach utilizing stabilometric parameters can effectively and accurately differentiate individuals with Parkinson’s disease (PD) from age-matched healthy older adults (HOAs). The robustness of our results is underpinned by a comprehensive validation strategy that involves repeated stratified data splitting and extensive bootstrap resampling, providing stable and generalizable performance estimates. This rigorous methodology enhances confidence in the clinical applicability of our findings and supports their potential use in future diagnostic and monitoring tools. Our optimized Ensemble Voting classifier achieved a high mean accuracy of approximately 90.5% and an excellent mean AUC-ROC of 0.9735, indicating strong discriminative power. Key stabilometric features, particularly those related to anteroposterior sway velocity with eyes open (V-AP (mm/s)) and total sway path with eyes closed (TOD_EC (mm)), were identified as robust differentiators, aligning with both univariate statistical significance and high importance in multivariate machine learning models. These findings underscore the potential of quantitative postural assessment combined with advanced analytics as an objective tool to support the clinical distinction between PD and healthy aging. Future research should focus on validating these models in larger, diverse cohorts and exploring their utility in tracking disease progression and response to therapeutic interventions.

## Figures and Tables

**Table 1:** Anthropometric characteristics of participants and classification of individuals with Parkinson’s disease (PD) according to the UPDRS and Hoehn and Yahr (H&Y) scale.

Characteristics	HOAs (*n* = 37)	PD (*n* = 26)
women	30 (81%)	10 (39%)
men	7 (19%)	16 (61%)
age, years (SD)	70 (6.8)	66 (2.9)
BMI (SD)	27 (5.3)	25 (4.6)
HOEHN and YAHR (SD)	-	2.5 (0.3)
UPDRS-III (SD)	-	21.2 (15.9)

Legend: SD, standard deviation; BMI: Body Mass Index; UPDRS-III: Unified Parkinson’s Disease Rating Scale. PD: Parkinson’s Disease; HOAs: Healthy Older Adults.

**Table 2:** Descriptive Statistics (median [IQR]) and Univariate Test Results.

Descriptive Statistics (Median [IQR])			Univariate Test Results
Parameter	HOA Median [IQR]	PD Median [IQR]	Bonferroni *p*
TOD (mm)	200 [52.5]	173.7 [167.8]	1.000
Std-AP (mm)	1.41 [0.46]	0.90 [0.95]	0.476
Std-ML (mm)	0.68 [0.35]	0.67 [0.77]	1.000
RMS-AP (mm)	1.40 [0.46]	0.90 [0.92]	0.467
RMS-ML (mm)	0.68 [0.34]	0.67 [0.76]	1.000
DA-AP (mm)	7.08 [2.13]	4.51 [4.71]	0.167
DA-ML (mm)	3.58 [1.43]	3.05 [4.31]	1.000
**V-AP (mm/s)**	**4.73 [2.48]**	**2.14 [3.24]**	0.008 *
V-ML (mm/s)	2.70 [1.10]	2.49 [1.67]	1.000
Area (mm^2^)	13.68 [9.92]	7.39 [20.42]	1.000
V-T (mm/s)	6.19 [2.29]	3.75 [4.81]	0.354
LF-AP (mm^2^/Hz)	1.34 [0.82]	0.68 [1.17]	0.353
MF-AP (mm^2^/Hz)	2.11 [1.41]	1.08 [1.55]	0.399
HF-AP (mm^2^/Hz)	0.63 [0.48]	0.58 [0.58]	1.000
LF-ML (mm^2^/Hz)	0.47 [0.45]	0.48 [0.75]	1.000
MF-ML (mm^2^/Hz)	1.35 [0.95]	0.90 [1.63]	1.000
HF-ML (mm^2^/Hz)	0.69 [0.53]	0.51 [0.84]	1.000
**TOD_EC (mm)**	**186.9 [90.6]**	**542.7 [531.1]**	0.001 *
Std-AP_EC (mm)	1.10 [0.69]	2.03 [2.23]	0.415
Std-ML_EC (mm)	0.79 [0.36]	1.03 [0.66]	1.000
RMS-AP_EC (mm)	1.10 [0.69]	2.02 [2.22]	0.399
RMS-ML_EC (mm)	0.79 [0.35]	1.02 [0.65]	1.000
DA-AP_EC (mm)	4.74 [2.60}	7.92 [9.36]	0.235
DA-ML_EC (mm)	3.79 [2.10]	4.67 [3.10]	1.000
V-AP_EC (mm/s)	1.53 [1.01]	1.61 [0.89]	1.000
V-ML_EC (mm/s)	2.27 [0.73]	1.94 [1.69]	1.000
Area_EC (mm^2^)	13.12 [10.78]	20.32 [53.59]	1.000
V-T_EC (mm/s)	3.13 [1.23]	2.95 [1.96]	1.000
LF-AP_EC (mm^2^/Hz)	1.06 [0.93]	2.95 [3.66]	1.000
MF-AP_EC (mm^2^/Hz)	1.97 [1.34]	1.85 [2.70]	1.000
HF-AP_EC (mm^2^/Hz)	0.75 [0.74]	0.71 [1.26]	1.000
LF-ML_EC (mm^2^/Hz)	0.44 [0.48]	1.15 [1.61]	1.000
MF-ML_EC (mm^2^/Hz)	1.63 [1.18]	1.73 [2.52]	1.000
HF-ML_EC (mm^2^/Hz)	0.79 [0.52]	0.81 [0.84]	1.000

Legend: PD, Parkinson’s Disease; HOAs: Healthy Older Adults. TOD: Total Oscillation Displacement; Std-AP, Std-ML, mm: Standard Deviation of COP displacement in AP and ML directions; RMS-AP, RMS-ML, mm: Root Mean Square of COP displacement in AP and ML directions; DA-AP, DA-ML, mm: Displacement Amplitude (Peak-to-peak) of COP in AP and ML directions; V-AP, V-ML, mm/s: the mean Velocity of COP in AP and ML directions; V-T, mm/s: Total Mean Velocity of COP. LF-AP, MF-AP, HF-AP, LF-ML, MF-ML, HF-ML: Frequency-domain variables, low frequency, medium frequency, and high frequency in the AP and ML directions. EC: Eyes-closed condition (e.g., TOD_EC, Std-AP_EC, etc.); * significant *p*-values.

**Table 3:** Mean [95% Confidence Interval] Performance Metrics of Tuned Machine Learning Models for HOA vs. PD Classification.

Model	Accuracy	F1 (PD)	F1 (HOAs)	AUC ROC	Sensitivity (PD)	Specificity (PD)	Precision (PD)	Precision (HOAs)
Random Forest	0.8912 [0.8079:0.9474]	0.8716 [0.7675:0.9412]	0.9052 [0.8318:0.9551]	0.9568 [0.8648:1.0000]	0.8917 [0.7500:1.0000]	0.8909 [0.8182:0.9682]	0.8587 [0.7597:0.9611]	0.9241 [0.8235:1.0000]
Gradient Boosting	0.9018 [0.8421:1.0000]	0.8772 [0.7692:1.0000]	0.9172 [0.8615:1.0000]	0.9652 [0.9205:1.0000]	0.8583 [0.6250:1.0000]	0.9333 [0.8182:1.0000]	0.9121 [0.7856:1.0000]	0.9072 [0.7857:1.0000]
SVM	0.8772 [0.7895:0.9474]	0.8669 [0.7778:0.9412]	0.8856 [0.8000:0.9551]	0.9591 [0.9017:1.0000]	0.9417 [0.8750:1.0000]	0.8303 [0.7273:0.9682]	0.8076 [0.7000:0.9611]	0.9536 [0.8889:1.0000]
Ensemble Voting	0.9053 [0.8079:1.0000]	0.8867 [0.7675:1.0000]	0.9181 [0.8318:1.0000]	0.9727 [0.9244:1.0000]	0.8833 [0.7500:1.0000]	0.9212 [0.8182:1.0000]	0.8978 [0.7597:1.0000]	0.9194 [0.8235:1.0000]

Legend: SVM: Support Vector Machine; AUC ROC: Area Under the ROC Curve; HOAs: Healthy Older Adults; PD: Parkinson’s disease.

**Table 4:** Representative Classification Report for the Tuned Ensemble Voting Model (HOAs vs. PD—first split (random seed = 0)).

Representative Classification Report for the Tuned Ensemble Voting Model (HOAs vs. PD).					
Class	Precision	F1-score	Support	Sensitivity	Specificity
HOA	0.9091	0.9091	11	0.9091	0.8750
PD	0.8750	0.8750	8	0.8750	0.9091

HOAs: Healthy Older Adults; PD: Parkinson’s disease.

## Data Availability

Data used in this work are available at the request of the corresponding author.

## Supplementary Materials

The following supporting information can be downloaded at: https://www.mdpi.com/article/10.3390/neurolint17090133/s1, Table S1. Shapiro–Wilk normality tests for all 34 stabilometric variables by group (HOA, PD), with *p*-values. Table S2. Univariate group comparisons for all 34 variables: medians [IQR], raw *p*-values, and multiplicity-adjusted *p*-values (Bonferroni and Benjamini–Hochberg FDR). Table S3. Paired Wilcoxon signed-rank tests comparing performance with vs. without SMOTE across the same 15 stratified splits, for each classifier (RF, GB, SVM, Ensemble) and metric (accuracy, F1 by class, AUC ROC, sensitivity, specificity); FDR-adjusted *p*-values reported. Table S4. Sensitivity analysis with SMOTE: mean (95% CI) performance metrics across 15 splits, formatted to mirror Table 3 in the main text (No-SMOTE).
