# Supplementary material for: High-Accuracy Classification of Parkinson’s Disease Using Ensemble Machine Learning and Stabilometric Biomarkers
Source: Neurol Int. 2025 Aug 26;17(9):133. doi: 10.3390/neurolint17090133 (PMC12472432; doi:10.3390/neurolint17090133)
Supplement: Supplementary file 1 [file neurolint-17-00133-s001.zip › neurolint-3769784-supplementary.pdf]

neurology international

MDPI

Supplement Tables

Supplementary *Table S1*: Univariate Test Results (p-values and corrected p-values):

Univariate Test Results (p-values and corrected p-values):							
**Parameter**	**Test Type**	**Statistic**	**Original p**	**Bonferroni p**	**FDR (BH) p**	**Sig (Bonf)**	**Sig (FDR)**
TOD (mm)	Mann-Whitney U	549.00	0.3460	1.000	0.5882	False	False
Std-AP (mm)	Mann-Whitney U	657.50	0.0140	0.4759	0.0433	False	True
Std-ML (mm)	Mann-Whitney U	466.00	0.8395	10.000	0.9045	False	False
RMS-AP (mm)	Mann-Whitney U	658.00	0.0137	0.4668	0.0433	False	True
RMS-ML (mm)	Mann-Whitney U	465.00	0.8286	1.000	0.9045	False	False
DA-AP (mm)	Mann-Whitney U	683.00	0.0049	0.1668	0.0433	False	True
DA-ML (mm)	Mann-Whitney U	536.00	0.4467	1.000	0.6904	False	False
V-AP (mm/s)	Mann-Whitney U	745.50	0.0002	0.0078	0.0039	True	True
V-ML (mm/s)	Mann-Whitney U	538.00	0.4302	1.000	0.6904	False	False
Area (mm²)	Mann-Whitney U	616.00	0.0604	1.000	0.1413	False	False
V-T (mm/s)	Mann-Whitney U	665.00	0.0104	0.3540	0.0433	False	True
LF-AP (mm²/Hz)	Mann-Whitney U	665.00	0.0104	0.3533	0.0433	False	True
MF-AP (mm²/Hz)	Mann-Whitney U	662.00	0.0117	0.3989	0.0433	False	True
HF-AP (mm²/Hz)	Mann-Whitney U	573.00	0.2013	1.000	0.3783	False	False
LF-ML (mm²/Hz)	Mann-Whitney U	469.00	0.8724	1.000	0.9045	False	False
MF-ML (mm²/Hz)	Mann-Whitney U	533.00	0.4721	1.000	0.6979	False	False
HF-ML (mm²/Hz)	Mann-Whitney U	518.00	0.6103	1.000	0.8045	False	False
TOD_EC (mm)	Mann-Whitney U	183.00	0.0000	0.0011	0.0011	True	True
Std-AP_EC (mm)	Mann-Whitney U	301.00	0.0122	0.4146	0.0433	False	True
Std-ML_EC (mm)	Mann-Whitney U	391.00	0.2114	1.000	0.3783	False	False
RMS-AP_EC (mm)	Mann-Whitney U	300.00	0.0117	0.3986	0.0433	False	True

RMS-ML_EC (mm)	Mann-Whitney U	390.00	0.2063	1.000	0.3783	False	False
DA-AP_EC (mm)	Mann-Whitney U	287.00	0.0069	0.2347	0.0433	False	True
DA-ML_EC (mm)	Mann-Whitney U	385.50	0.1847	1.000	0.3783	False	False
V-AP_EC (mm/s)	Mann-Whitney U	509.00	0.7010	1.000	0.8463	False	False
V-ML_EC (mm/s)	Mann-Whitney U	517.50	0.6152	1.000	0.8045	False	False
Area_EC (mm^2)	Mann-Whitney U	347.00	0.0624	1.000	0.1413	False	False
V-T_EC (mm/s)	Mann-Whitney U	522.50	0.5670	1.000	0.8033	False	False
LF-AP_EC (mm^2/Hz)	Mann-Whitney U	327.00	0.0321	1.000	0.0899	False	False
MF-AP_EC (mm^2/Hz)	Mann-Whitney U	455.00	0.7218	1.000	0.8463	False	False
HF-AP_EC (mm^2/Hz)	Mann-Whitney U	490.00	0.9055	1.000	0.9055	False	False
LF-ML_EC (mm^2/Hz)	Mann-Whitney U	329.00	0.0344	1.000	0.0899	False	False
MF-ML_EC (mm^2/Hz)	Mann-Whitney U	451.50	0.6856	1.000	0.8463	False	False
HF-ML_EC (mm^2/Hz)	Mann-Whitney U	492.50	0.8779	1.000	0.9045	False	False

Supplementary *Table S2*: Shapiro-Wilk Normality Test Results (p-values):

**Parameter**	**HOA (p)**	**PD (p)**	**Levene Test (p)**
TOD (mm)	0.0000	0.0128	0.0416
Std-AP (mm)	0.0000	0.0120	0.0910
Std-ML (mm)	0.0000	0.0000	0.1186
RMS-AP (mm)	0.0000	0.0121	0.0938
RMS-ML (mm)	0.0000	0.0000	0.1188
DA-AP (mm)	0.0000	0.0025	0.0265
DA-ML (mm)	0.0000	0.0000	0.0752
V-AP (mm/s)	0.3234	0.0000	0.0971
V-ML (mm/s)	0.1651	0.0024	0.0111
Area (mm^2)	0.0000	0.0000	0.3777
V-T (mm/s)	0.3395	0.0033	0.0060
LF-AP (mm^2/Hz)	0.8556	0.0003	0.1632
MF-AP (mm^2/Hz)	0.0000	0.0019	0.9246
HF-AP (mm2/Hz)	0.0003	0.0000	0.0304
LF-ML (mm^2/Hz)	0.0001	0.0013	0.0754
MF-ML (mm^2/Hz)	0.0000	0.0000	0.4038

HF-ML (mm^2/Hz)	0.0000	0.0000	0.0690
TOD_EC (mm)	0.0000	0.0005	0.0001
Std-AP_EC (mm)	0.0000	0.1198	0.0001
Std-ML_EC (mm)	0.0000	0.0016	0.0575
RMS-AP_EC (mm)	0.0000	0.1196	0.0001
RMS-ML_EC (mm)	0.0000	0.0015	0.0566
DA-AP_EC (mm)	0.0001	0.0771	0.0001
DA-ML_EC (mm)	0.0000	0.0000	0.1183
V-AP_EC (mm/s)	0.0085	0.0000	0.0495
V-ML_EC (mm/s)	0.0022	0.0004	0.0187
Area_EC (mm^2)	0.0000	0.0001	0.0152
V-T_EC (mm/s)	0.2834	0.0001	0.0183
LF-AP_EC (mm^2/Hz)	0.0016	0.0014	<0.0001
MF-AP_EC (mm^2/Hz)	0.0000	0.0021	0.0729
HF-AP_EC (mm^2/Hz)	0.0001	0.0000	0.0908
LF-ML_EC (mm^2/Hz)	0.0000	0.1067	0.0017
MF-ML_EC (mm^2/Hz)	0.0000	0.0003	0.0482
HF-ML_EC (mm^2/Hz)	0.0000	0.0000	0.0466

Supplementary *Table S3*: Comparison between analysis SMOTE and non-SMOTE pipelines performed equivalently for all models. Paired Wilcoxon signed-rank tests across the same 15 paired stratified splits.

model	metric	n_pairs	wilcoxon_stat	p_value	p_value_fdr
Random Forest	accuracy	15	15	0.367881	0.588609
Random Forest	f1_pos	15	18.5	0.634992	0.845088
Random Forest	f1_neg	15	13.5	0.285474	0.527029
Random Forest	auc	15	18	0.181666	0.435998
Random Forest	spec_pos	15	2	0.256839	0.513679
Random Forest	spec_neg	15	10.5	1	1
Random Forest	sens_pos	15	10.5	1	1
Random Forest	sens_neg	15	2	0.256839	0.513679
Gradient Boosting	accuracy	15	15	0.669028	0.845088
Gradient Boosting	f1_pos	15	16	0.779435	0.935321
Gradient Boosting	f1_neg	15	12	0.400814	0.601221
Gradient Boosting	auc	15	27.5	0.366432	0.588609
Gradient Boosting	spec_pos	15	0	0.0656	0.222039
Gradient Boosting	spec_neg	15	0	0.033895	0.222039
Gradient Boosting	sens_pos	15	0	0.033895	0.222039
Gradient Boosting	sens_neg	15	0	0.0656	0.222039
SVM	accuracy	15	0	0.083265	0.222039
SVM	f1_pos	15	0	0.083265	0.222039
SVM	f1_neg	15	0	0.083265	0.222039
SVM	auc	15	18	0.59134	0.834833
SVM	spec_pos	15	0	0.083265	0.222039

SVM	spec_neg	15	0	NA	1
SVM	sens_pos	15	0	NA	1
SVM	sens_neg	15	0	0.083265	0.222039

Legend: p_value_ fdr: paired Wilcoxon signed-rank tests with Benjamini–Hochberg false discovery rate correction ; SVS: Support Vector Machine; auc: Area Under the ROC Curve; spec_pos: specificity positive; spec_neg: specificity negative; sens_pos: sensitivity positive; sens_negative: sensitivity.

Supplementary *Table S4:* Mean (95% Confidence Interval) Performance Metrics of Tuned Machine Learning Models for HOA vs. PD Classification for models after adjusting for moderate class imbalance using SMOTE.

Model	Accuracy	F1 (PD)	F1 (HOA)	AUC ROC	Sensitivity (PD)	Specificity (PD)	Precision (PD)	Precision (HOA)
Random Forest	0.8807 [0.8421 : 0.9474]	0.8620 [0.8000 : 0.9412]	0.8946 [0.8571 : 0.9524]	0.9458 [0.8506 : 0.9960]	0.8917 [0.7500 : 1.0000]	0.8727 [0.8182 : 0.9091]	0.8380 [0.7778 : 0.8889]	0.9202 [0.8333 : 1.0000]
Gradient Boosting	0.8982 [0.8079 : 0.9816]	0.8845 [0.8000 : 0.9794]	0.9075 [0.8003 : 0.9848]	0.9568 [0.8449 : 1.0000]	0.9083 [0.7500 : 1.0000]	0.8909 [0.6682 : 1.0000]	0.8756 [0.6879 : 1.0000]	0.9365 [0.8378 : 1.0000]
SVM	0.8877 [0.7895 : 0.9474]	0.8773 [0.7778 : 0.9412]	0.8961 [0.8000 : 0.9551]	0.9583 [0.9057 : 1.0000]	0.9417 [0.8750 : 1.0000]	0.8485 [0.7273 : 0.9682]	0.8253 [0.7000 : 0.9611]	0.9536 [0.8889 : 1.0000]
Ensemble Voting	0.9053 [0.8079 : 1.0000]	0.8866 [0.7675 : 1.0000]	0.9181 [0.8318 : 1.0000]	0.9697 [0.9244 : 1.0000]	0.8833 [0.7500 : 1.0000]	0.9212 [0.8182 : 1.0000]	0.8987 [0.7597 : 1.0000]	0.9199 [0.8235 : 1.0000]

Legend: SVM: Support Vector Machine; AUC ROC: Area Under the ROC Curve; HOA: Healthy Older Adults; PD: Parkinson's disease.
